# Trends in socioeconomic inequalities in amenable mortality in urban areas of Spanish cities, 1996–2007

**DOI:** 10.1186/1471-2458-14-299

**Published:** 2014-04-01

**Authors:** Andreu Nolasco, José Antonio Quesada, Joaquín Moncho, Inmaculada Melchor, Pamela Pereyra-Zamora, Nayara Tamayo-Fonseca, Miguel Angel Martínez-Beneito, Oscar Zurriaga

**Affiliations:** 1 Unidad de Investigación de Análisis de la Mortalidad y Estadísticas Sanitarias. Departamento de Enfermería Comunitaria, Medicina Preventiva y Salud Pública e Historia de la Ciencia, Universidad de Alicante Campus de San Vicente del Raspeig s/n, Apartado 99, 03080 Alicante, España; 2 Registro de Mortalidad de la Comunidad Valenciana. Servicio de Estudios Epidemiológicos y Estadísticas Sanitarias, Subdirección General de Epidemiología y Vigilancia de la Salud. Conselleria de Sanitat, Plaza de España 6, 03010 Alicante, España; 3 Área de Desigualdades en Salud. FISABIO-CSISP. Conselleria de Sanitat, Avenida de Cataluña, 21, 46020 Valencia, España; 4 Servicio de Estudios Epidemiológicos y Estadísticas Sanitarias, Subdirección General de Epidemiología y Vigilancia de la Salud. Conselleria de Sanitat, Avenida de Cataluña, 21, 46020 Valencia, España; 5 Consorcio de Investigación Biomédica en Red de Epidemiología y Salud Pública (CIBERESP), Instituto de Salud Carlos III Melchor Fernández Almagro, 3-5 28029 Madrid, España

**Keywords:** Inequalities in health, Equity in healthcare, Amenable mortality, Small area analysis, Urban areas

## Abstract

**Background:**

While research continues into indicators such as preventable and amenable mortality in order to evaluate quality, access, and equity in the healthcare, it is also necessary to continue identifying the areas of greatest risk owing to these causes of death in urban areas of large cities, where a large part of the population is concentrated, in order to carry out specific actions and reduce inequalities in mortality. This study describes inequalities in amenable mortality in relation to socioeconomic status in small urban areas, and analyses their evolution over the course of the periods 1996–99, 2000–2003 and 2004–2007 in three major cities in the Spanish Mediterranean coast (Alicante, Castellón, and Valencia).

**Methods:**

All deaths attributed to amenable causes were analysed among non-institutionalised residents in the three cities studied over the course of the study periods. Census tracts for the cities were grouped into 3 socioeconomic status levels, from higher to lower levels of deprivation, using 5 indicators obtained from the 2001 Spanish Population Census. For each city, the relative risks of death were estimated between socioeconomic status levels using Poisson’s Regression models, adjusted for age and study period, and distinguishing between genders.

**Results:**

Amenable mortality contributes significantly to general mortality (around 10%, higher among men), having decreased over time in the three cities studied for men and women. In the three cities studied, with a high degree of consistency, it has been seen that the risks of mortality are greater in areas of higher deprivation, and that these excesses have not significantly modified over time.

**Conclusions:**

Although amenable mortality decreases over the time period studied, the socioeconomic inequalities observed are maintained in the three cities. Areas have been identified that display excesses in amenable mortality, potentially attributable to differences in the healthcare system, associated with areas of greater deprivation. Action must be taken in these areas of greater inequality in order to reduce the health inequalities detected. The causes behind socioeconomic inequalities in amenable mortality must be studied in depth.

## Background

Avoidable mortality, as an indicator for the quality of healthcare services, was first introduced by Rustein in 1976, who presented the first theoretical study on this issue, where he proposed a list of unnecessary diseases and disabilities or unnecessary untimely deaths, based on the assertion that if health services had acted correctly, they would have been prevented or delayed [1]. Its definition and conceptualisation, as well as the list of causes of death included therein, has evolved over time adapting to medical and technological advances [2-9].

Avoidable mortality is divided into two groups, according to the characteristics of the healthcare intervention 1) Amenable mortality: related with secondary prevention and directly with healthcare intervention, in other words access to healthcare, advice, diagnosis, or treatment: and 2) Preventable mortality: related with primary prevention, lifestyle habits, intervention programmes, etc. [10].

Given that the quality of healthcare and fair access to it must be considered objectives of health policy, amenable mortality can be considered an indicator of potential weaknesses in the healthcare system, in order to study these weaknesses in greater depth and has been used for decades to show the positive impact of healthcare on the health of the population in many industrialised countries [5,11].

Studies conducted in different countries have related certain population socioeconomic indicators with avoidable mortality and, in particular, with amenable, pointing to higher mortality rates among the more underprivileged groups [12-23]. These inequalities are in themselves a risk factor for the health of the population, and they must be studied in order to identify the most vulnerable groups and regions, and be able to carry out specific interventions [24].

In recent decades, improvements in living conditions and growing accessibility to healthcare assistance have led to a reduction in premature mortality and, therefore, avoidable mortality, both amenable and preventable. Various studies evaluating trends in avoidable mortality over time in specific regions or groups have shown this decline [5,7,25-30]. Some studies that have linked this temporal evolution with socioeconomic inequalities have suggested that socioeconomic inequalities in avoidable mortality have remained and even increased in recent years [12,18,20,23,31]. Other studies that have analysed avoidable mortality in small areas, or which combine this analysis with a study of the association observed with indicators of inequality, have demonstrated an important link between the most depressed areas and those with the highest levels of mortality [14,19,32-34].

Research continues into indicators such as preventable and amenable mortality in order to evaluate quality, access, and equity in the healthcare system [9,35-37]. It is also necessary to continue identifying the areas of greatest risk owing to these causes of death in urban areas of large cities, where a large part of the population is concentrated, in order to carry out specific actions and reduce inequalities in mortality. In Spain, to date, there have been no studies conducted to ascertain the evolution of amenable avoidable mortality and its association with socioeconomic status in small areas of large cities.

The objective of this study is to analyse the temporal evolution of amenable mortality between 1996 and 2007 and its association with socioeconomic status in small areas (census tracts) of the three largest cities, province capitals, of the Comunitat Valenciana (Spain): Alicante, Castellón, and Valencia.

## Methods

This study was performed within the framework of the MEDEA project [38,39]. This is a transversal ecological study of amenable mortality in the cities of Alicante, Castellón, and Valencia in the period (P) 1996–2007. These cities are located on the south east coast of Spain (Mediterranean coast), in the region of the Comunitat Valenciana (CV). All the deaths corresponded to non-institutionalised individuals residing in these three cities during the study period. The deaths were georeferenced by census tract and were obtained from databases used with permission of the CV Death Register Office. The causes of death between 1996 and 1998 were encoded in accordance with the International Classification of Diseases, 9th Version (ICD-9), and the deaths that took place between 1999 and 2007 were classified using the 10th version (ICD-10).

The causes of amenable deaths analysed in the study were those proposed by Nolte and McKee, see Table 1, and following the criterion defined by these authors, only 50% of the deaths due to Ischaemic Heart Disease were included [7,11].

**Table 1:** Frequencies and percentages (by city and sex), classification codes ICD-9 and ICD-10, and age range for the amenable causes studied

	**CAUSE**	**ICD-9**	**ICD-10**	**AGE**	**Sex**	**Alicante**		**Castellón**		**Valencia**	
						**n**	**%**	**n**	**%**	**n**	**%**
1	Intestinal infections				M	0	0.0	1	0.1	2	0.0
		002-009	A00-A09	0-14	F	0	0.0	0	0.0	0	0.0
2	Tuberculosis	010-018,	A15-A19,		M	25	1.6	11	1.4	41	0.9
		137	B90	0-74	F	4	0.3	4	0.6	12	0.3
3	Other infections (diphtheria, tetanus, poliomyelitis)	032	A36, A35,		M	0	0.0	0	0.0	2	0.0
		037, 045	A80	0-74	F	0	0.0	0	0.0	0	0.0
4	Whooping cough				M	0	0.0	0	0.0	0	0.0
		033	A37	0-14	F	0	0.0	0	0.0	0	0.0
5	Septicaemia				M	58	3.7	13	1.7	115	2.5
		038	A40-A41	0-74	F	22	1.8	9	1.4	70	2.0
6	Measles				M	0	0.0	0	0.0	0	0.0
		055	B05	1-14	F	0	0.0	0	0.0	0	0.0
7	Malignant neoplasm of colon and rectum				M	269	17.3	144	18.8	846	18.7
		153-154	C18-C21	0-74	F	201	16.2	114	18.1	508	14.4
8	Malignant neoplasm of skin				M	3	0.2	0	0.0	14	0.3
		173	C44	0-74	F	3	0.2	1	0.2	4	0.1
9	Malignant neoplasm of breast				M	2	0.1	0	0.0	4	0.1
		174	C50	0-74	F	317	25.5	140	22.2	950	26.9
10	Malignant neoplasm of cervix uteri				M	0	0.0	0	0.0	0	0.0
		180	C53	0-74	F	38	3.1	21	3.3	118	3.3
11	Malignant neoplasm of cervix uteri and body uterus				M	0	0.0	0	0.0	0	0.0
		179, 182	C54-C55	0-44	F	2	0.2	0	0.0	4	0.1
12	Malignant neoplasm of testis				M	3	0.2	1	0.1	7	0.2
		186	C62	0-74	F	0	0.0	0	0.0	0	0.0
13	Hodgkin’s disease				M	6	0.4	3	0.4	13	0.3
		201	C81	0-74	F	2	0.2	4	0.6	18	0.5
14	Leukaemia				M	12	0.8	10	1.3	50	1.1
		204-208	C91-C95	0-44	F	11	0.9	5	0.8	23	0.7
15	Diseases of the thyroid				M	3	0.2	1	0.1	1	0.0
		240-246	E00-E07	0-74	F	6	0.5	1	0.2	13	0.4
16	Diabetes				M	7	0.5	5	0.7	19	0.4
		250	E10-E14	0-49	F	2	0.2	1	0.2	6	0.2
17	Epilepsy				M	8	0.5	4	0.5	22	0.5
		345	G40-G41	0-74	F	4	0.3	3	0.5	21	0.6
18	Chronic rheumatic heart disease				M	21	1.4	10	1.3	70	1.5
		393-398	I05-I09	0-74	F	36	2.9	34	5.4	148	4.2
19	Hypertensive disease		I10-I13,		M	37	2.4	23	3.0	99	2.2
		401-405	I15	0-74	F	31	2.5	20	3.2	89	2.5
20	Ischaemic heart disease: 50% of deaths				M	510	32.9	234	30.6	1366	30.2
		410-414	I20-I25	0-74	F	185	14.9	79	12.5	394	11.2
21	Cerebrovascular diseases				M	352	22.7	205	26.8	1059	23.4
		430-438	I60-I69	0-74	F	238	19.2	116	18.4	719	20.4
22	All respiratory diseases	460-479,	J00-J09,		M	0	0.0	1	0.1	3	0.1
		488-519	J20-J99	1-14	F	0	0.0	0	0.0	3	0.1
23	Influenza				M	0	0.0	0	0.0	2	0.0
		487	J10-J11	0-74	F	0	0.0	0	0.0	7	0.2
24	Pneumonia				M	84	5.4	35	4.6	351	7.8
		480-486	J12-J18	0-74	F	34	2.7	17	2.7	157	4.4
25	Peptic ulcer				M	24	1.5	6	0.8	48	1.1
		531-533	K25-K27	0-74	F	7	0.6	4	0.6	13	0.4
26	Appendicitis				M	1	0.1	2	0.3	8	0.2
		540-543	K35-K38	0-74	F	1	0.1	1	0.2	4	0.1
27	Abdominal hernia				M	1	0.1	3	0.4	8	0.2
		550-553	K40-K46	0-74	F	1	0.1	3	0.5	15	0.4
28	Cholelithiasis and cholecystitis				M	8	0.5	9	1.2	30	0.7
		574-575.1	K80-K81	0-74	F	6	0.5	5	0.8	22	0.6
29	Nephritis and nephrosis		N00-N07,		M	66	4.3	22	2.9	188	4.2
		580-589	N17-N19, N25-N27	0-74	F	46	3.7	21	3.3	97	2.7
30	Benign prostatic hyperplasia				M	1	0.1	0	0.0	2	0.0
		600	N40	0-74	F	0	0.0	0	0.0	0	0.0
31	Maternal death				M	0	0.0	0	0.0	0	0.0
		630-676	O00-O99	All	F	2	0.2	0	0.0	3	0.1
32	Congenital cardiovascular anomalies				M	15	1.0	9	1.2	52	1.1
		745-747	Q20-Q28	0-74	F	15	1.2	9	1.4	39	1.1
33	Perinatal deaths		P00-P96,		M	34	2.2	12	1.6	101	2.2
		760-779	A33, A34	All	F	26	2.1	17	2.7	72	2.0
34	Misadventures to patients	E870-E876,	Y60-Y69,		M	1	0.1	0	0.0	4	0.1
		E878-E879	Y83-Y84	All	F	2	0.2	1	0.2	2	0.1
	TOTAL AMENABLE				M	1551	100.0	764	100.0	4527	100.0
					F	1242	100.0	630	100.0	3531	100.0

The unit of analysis for each city was the Census Tract (CT). CT urban structure of the cities belonging to 2001 was used. According to the population census for the year 2001, the city of Alicante had 284,580 inhabitants, Castellón 147,667, and Valencia 737,219. The number of CTs studied were 215 for Alicante, 95 in Castellón, and 553 in Valencia. The average population size for the CTs studied was 1355 inhabitants.

In each city, the socioeconomic status (SES) of each CT was established using the following indicators (in percentages):

### Unemployed

Percentage of people aged 16 or over without work (unemployed and those looking for work for the first time), in relation to the total active population.

### Low level of education

Percentage of people aged 16 or over who, according to the lists from the National Institute of Statistics, cannot read or write, or can read and write but have fewer than 5 years’ schooling, or went to school for 5 or more years but did not complete their primary studies, in relation to the total population aged 16 or over.

### Low level of education among young people

Percentage of people aged 16 to 29 with a low level of education in relation to the total population aged 16 to 29.

### Manual workers

Percentage of people aged 16 or over who have a manual job (employees in the following sectors: services, agriculture, fishing, artisan work, specialist workers in manufacturing industries, construction, mining, installation operators, and non-specialist workers) in relation to the total number of people aged 16 or over in employment.

### Temporary workers

Percentage of people aged 16 or over in temporary employment (part-time self-employed workers, temporary workers), in relation to the total number of people aged 16 or over in employment.

These indicators were used previously in the MEDEA project. Their capacity to classify census tracts by socioeconomic status has been demonstrated in a prior study [34]. On the basis of these indicators, three levels were selected for the SES: SES1 (most privileged socioeconomic status) includes all CTs with values below the 25th percentile in the 5 indicators, SES3 (least privileged socioeconomic status) represents all CTs with values above the 75th percentile; the remaining CTs were included in SES2 (intermediate socioeconomic status). The databases of indicators were freely available and were obtained from the Population and Housing Census - 2001 of the Spanish National Statistics Institute.

**Figure 1:**
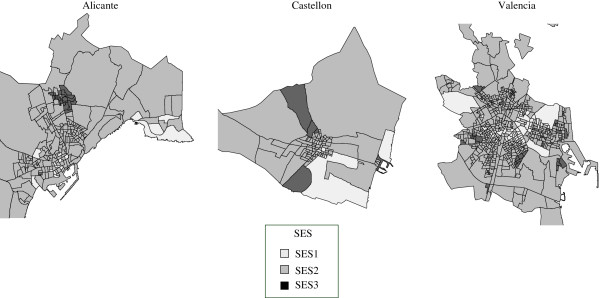
Geographical distribution of the Socioeconomic Status (SES) according to census tracts in Alicante, Castellón and Valencia.

Table 2 shows the distribution of CTs and population by SES. Figure 1 shows the geographical distribution of the CTs by SES for the three cities studied.

**Table 2:** Number of Census Tracts (CTs) and population of 2001 (Pop) by SES level, and frequencies and percentages of death due to amenable mortality (AM) in comparison to total mortality (TM), by sex, period, and city

**CITY**	**SES1**		**SES2**		**SES3**		**TYPE MORTAL**	**SEX**	**1996-99**		**2000-03**		**2004-07**		**1996-2007**	
	**CTs**	**Pop**	**CTs**	**Pop**	**CTs**	**Pop**			**n**	**%**	**n**	**%**	**n**	**%**	**n**	**%**
Alicante	15	21404	180	218224	20	22856	AM	M	551	11.8	525	10.7	475	9.8	1551	10.7
								F	471	11.6	393	9.5	378	8.7	1242	9.9
							TM	M	4682	100.0	4893	100.0	4854	100.0	14429	100.0
								F	4046	100.0	4158	100.0	4366	100.0	12570	100.0
Castellón	6	10266	84	118144	5	8418	AM	M	304	12.0	220	8.5	240	9.3	764	9.9
								F	230	10.9	191	8.6	209	9.3	630	9.6
							TM	M	2542	100.0	2598	100.0	2583	100.0	7723	100.0
								F	2107	100.0	2232	100.0	2253	100.0	6592	100.0
Valencia	53	62859	456	576146	44	49370	AM	M	1693	11.6	1502	10.6	1332	9.6	4527	10.6
								F	1351	10.3	1155	8.7	1025	7.4	3531	8.8
							TM	M	14578	100.0	14109	100.0	13931	100.0	42618	100.0
								F	13177	100.0	13293	100.0	13870	100.0	40340	100.0

The populations by CT, age and sex used to calculate mortality indices for the years studied were obtained with permission from the Register Office of the Valencian Institute of Statistics, which is responsible for compiling population statistics in this region.

To study the evolution of the risk of death over time, the data were classified into three time periods: 1996–1999 (P1), 2000–2003 (P2), and 2004–2007 (P3). Deaths were also grouped by age range: 0–49, 50–64, and 65–74 years of age.

The number and percentage of deaths were calculated for each cause, by sex, period, and city, along with the specific mortality rates (per 100,000 inhabitants). Age Standardised Rates (ASR) have been calculated for each city, sex, period, and SES, using the Spanish population of 2001 as the standard population. Poisson regression models were adjusted using the logarithm of the death rate to estimate the Relative Risks (RR) of death, and their corresponding intervals of confidence at 95%, for each level of the explanatory variables SES, period, and age group [40]. Interaction between SES and period, SES and age group, and age group and period was studied. Possible overdispersion of the models was controlled [41]. The statistical software package R version 2.12.2 was used for analysis. All the analyses were separated by sex.

### Ethical review

All data used in the analysis have been routinely collected by government statistical agencies, and were anonymized before they were supplied to us. Under these conditions, national regulations do not require ethical review of study proposals.

## Results

Amenable mortality represents approximately 10% of total mortality, for men and women, and for the three cities studied (Table 2). Among men, there were 1551 deaths in Alicante, 764 in Castellón and 4527 in Valencia. Among women, there were 1242, 630, and 3531 deaths in each city, respectively.

By time periods, for the three cities studied, there is a slight decline in amenable mortality from 12% in the first period to 10% in the third period, approximately (Table 2).

The most frequent cause of amenable mortality with regard to the total for amenable causes was ischaemic heart disease (IHD) for men, with 32.9% in Alicante, 30.7% in Castellón, and 30.2% in Valencia (Table 1). Among women, the most frequent cause of death was breast tumour, representing 22.2%, 19.7% and 26.9% in each city, respectively. The second most frequent amenable cause among men and women was cerebrovascular diseases in the three cities, with percentages between 17% and 23% among men, and 16% and 20% among women.

**Figure 2:**
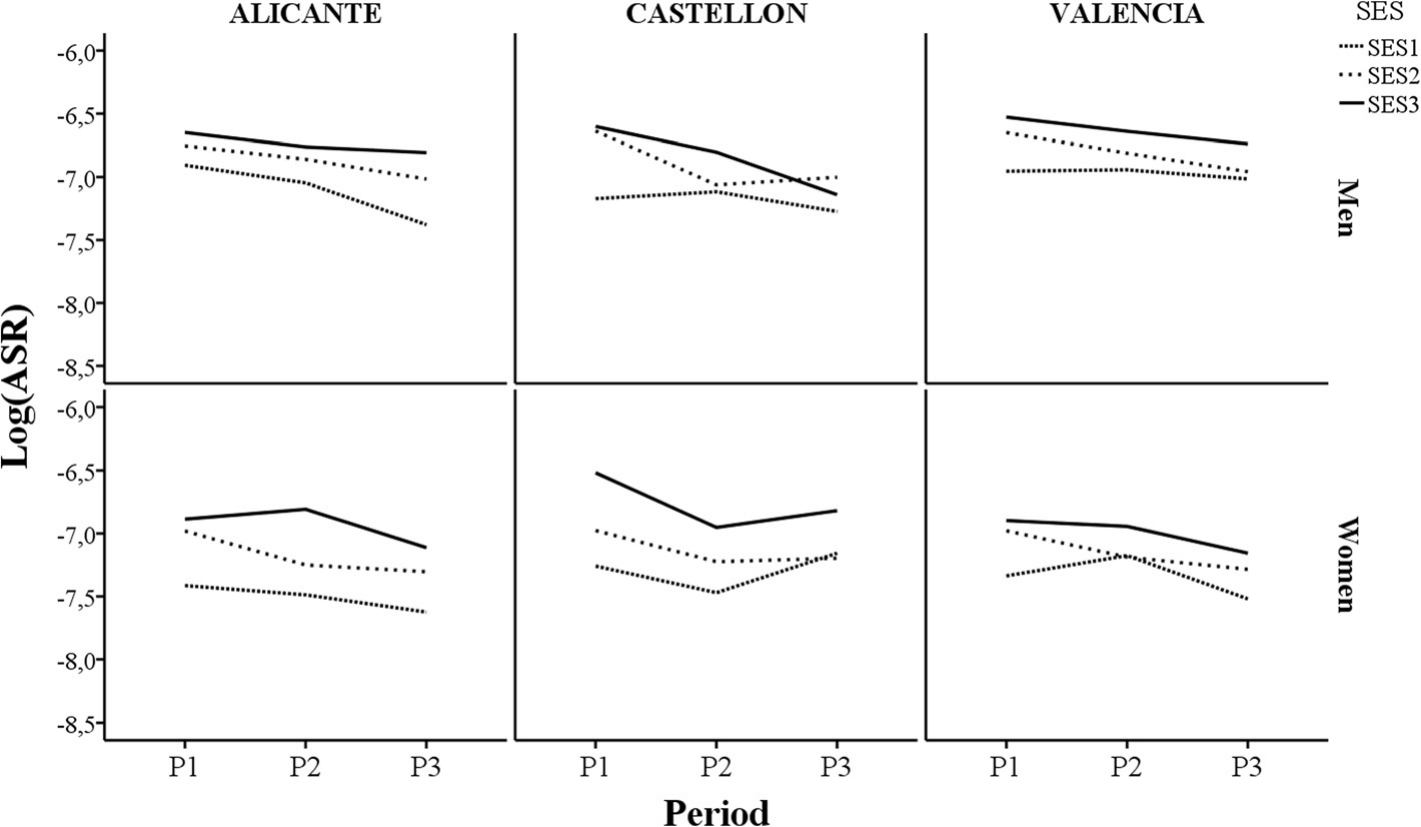
Evolution of Age Standardised Rate (Log(ARS)) by socioeconomic status (SES), city, and sex.

Table 3 shows the specific rates of amenable mortality by sex, period, and age. In overall, the specific rates of amenable mortality are higher in the least privileged group in the three periods, but there are some exceptions that suggest that the relative risk between the SES levels be evaluated globally. Figure 2 shows the evolution of the ASR (logASR) by SES level for each city, and sex. As can be observed, there is a decrease of the ASR from the first period to the last one, with differences depending on city and sex, in all cases, except for SES1 in Castellón for women. For men, the decrease of the ASR was slightly higher in SES1 in Alicante and higher in SES3 in Castellón and Valencia. The distance between SES1 and SES3 increased from the first period to the last one only in Alicante, and decreased the most in Castellón. For women, the decrease in the ASR was similar in SES1 and SES3 in Alicante and Valencia, whereas they decreased in SES3 and slightly increased in SES1 in Castellón. The distances between SES1 and SES3 were very similar in the first and last period, slightly lower in the last period in Castellón.

**Table 3:** Specific rates (x100.000) of amenable mortality by sex, period, age, socioeconomic status level (SES), and city

**MALE**										
**Period (years)**		**ALICANTE**			**CASTELLÓN**			**VALENCIA**		
		**0-49**	**50-64**	**65-74**	**0-49**	**50-64**	**65-74**	**0-49**	**50-64**	**65-74**
1996-99	SES1	13,1	131,4	717,7	24,4	112,1	420,2	19,1	162,2	568,6
	SES2	24,8	171,4	730,5	22,5	209,5	832,4	23,3	223,9	781,9
	SES3	13,4	214,4	877,8	8,0	272,6	881,5	33,2	259,9	819,7
2000-03	SES1	12,7	108,2	624,3	6,2	175,5	493,9	20,6	136,1	614,3
	SES2	17,5	162,0	680,2	15,6	115,6	575,7	21,7	177,7	673,8
	SES3	28,2	185,9	667,7	23,5	167,7	687,8	36,6	189,6	754,3
2004-07	SES1	9,9	80,4	437,4	17,1	64,5	482,2	23,1	121,2	550,8
	SES2	16,7	140,4	564,8	18,0	145,3	560,1	19,9	155,9	570,1
	SES3	23,7	223,7	583,4	18,0	247,0	257,1	26,6	267,8	565,7
**FEMALE**										
**Period (years)**		**ALICANTE**			**CASTELLÓN**			**VALENCIA**		
		**0-49**	**50-64**	**65-74**	**0-49**	**50-64**	**65-74**	**0-49**	**50-64**	**65-74**
1996-99	SES1	22,2	121,3	205,6	6,6	109,6	411,2	22,1	85,1	304,9
	SES2	20,9	145,1	466,1	19,5	133,6	496,0	25,0	141,1	448,5
	SES3	17,1	179,7	518,2	32,3	235,1	738,5	16,1	192,4	494,1
2000-03	SES1	9,1	50,0	361,0	18,0	89,2	252,3	27,4	105,0	342,1
	SES2	15,7	118,3	346,1	18,0	125,7	336,5	22,6	110,4	355,2
	SES3	27,9	121,9	613,6	16,2	182,0	462,2	18,0	175,5	468,3
2004-07	SES1	22,0	71,6	182,3	19,3	53,4	485,7	10,8	82,9	283,0
	SES2	15,3	117,4	316,9	19,0	100,3	388,2	20,5	110,5	306,5
	SES3	22,6	158,2	332,8	30,8	84,6	643,2	17,6	113,1	403,9

Table 4 shows the Relative Risks (RR) of death and their confidence intervals at 95% obtained using Poisson regression models with explanatory variables SES, age, and period. It cannot be stated that inequalities in the risks of death according to SES have changed significantly over the time periods studied, since there is no significant interaction between SES and period in any of the cases studied. For men, in Alicante, the risk of death is 1.5 (1.1-1.9) times higher when they are in the most underprivileged group SES3 in comparison with SES1, and the risk of death decreases in the third period in comparison with the first, RR = 0.8 (0.7-0.9). In Castellón, the risk of death decreases in P2 and P3 in comparison with P1. In this city, differences due to SES are not significant among men, although the values for relative risk among SES levels are similar to those obtained in the other two cities. In Valencia, the relative risk of death is 0.8 (0.7-0.8) in P3 and 0.9 (0.8-0.9) in P2 in comparison with P1, and the risk of death is 1.4 (1.2-1.6) times higher in SES3 than in SES1.

**Table 4:** Adjusted Relative Risks (RR) of death and confidence intervals at 95% for age, period, and socioeconomic status, according to city and sex

		RR of death - CI at 95% (*)	
		**MEN**	**WOMEN**
ALICANTE			
Age	00-49	1	1
	50-64	**8.2** (7.0-9.7)	**7.1** (6.0-8.4)
	65-74	**34.2** (29.6-39.5)	**21.1** (18.1-24.7)
Period	96-99	1	1
	00-03	0.9 (0.8-1.0)	**0.8** (0.7-0.9)
	04-07	**0.8** (0.7-0.9)	**0.7** (0.6-0.8)
SES	SES1	1	1
	SES2	1.3 (1.0-1.6)	**1.4** (1.1-1.8)
	SES3	**1.5** (1.1-1.9)	**1.8** (1.3-2.4)
CASTELLÓN			
Age	00-49	1	1
	50-64	**8.4** (6.7-10.5)	**6.3** (5.0-7.9)
	65-74	**34.2** (27.9-41.9)	**21.9** (17.8-27.0)
Period	96-99	1	1
	00-03	**0.7** (0.6-0.8)	**0.8** (0.6-0.9)
	04-07	**0.7** (0.6-0.8)	**0.8** (0.7-0.9)
SES	SES1	1	1
	SES2	1.3 (0.9-1.7)	1.1 (0.8-1.6)
	SES3	1.4 (0.9-2.1)	**1.7** (1.1-2.6)
VALENCIA			
Age	00-49	1	
	50-64	**8.3** (7.6-9.1)	(**)
	65-74	**29.8** (27.4-32.4)	
Period	96-99	1	1
	00-03	**0.9** (0.8-0.9)	**0.8** (0.8-0.9)
	04-07	**0.8** (0.7-0.8)	**0.7** (0.7-0.8)
SES	SES1	1	
	SES2	**1.2** (1.1-1.3)	(**)
	SES3	**1.4** (1.2-1.6)	
(**) 00-49	SES1		1
	SES2		1.1 (0.9-1.5)
	SES3		0.9 (0.6-1.3)
(**) 50-64	SES1		1
	SES2		**1.3** (1.0-1.6)
	SES3		**1.8** (1.3-2.4)
(**) 65-74	SES1		1
	SES2		1.2 (1.0-1.4)
	SES3		**1.5** (1.2-1.8)

(*) In bold, RR are statistically significant at 95%.

(**) Owing to the existence of significant interaction between age and SES for women, the RRs for the SES are shown in each of the age groups.

Among women, in the three cities, the risk of death decreases in P2 and P3 in comparison to P1. By SES, the risk of death in SES3 is 1.8 (1.3-2.4) times higher than for SES1 in Alicante, and 1.7 (1.1-2.6) times higher in Castellón. In Valencia, this risk is 1.8 (1.3-2.4) times higher in the 50–64 age group and 1.5 (1.2-1.8) times higher in the 65–74 age range, since there is a significant interaction between SES and age group.

## Discussion

Using data from three major Spanish cities, basic socioeconomic indicators of the educational and working environment have been used in this study to examine socioeconomic inequalities in amenable mortality and thus evaluate equity in the effectiveness of the health service. The existence of inequalities in amenable mortality should highlight the differences in the result of healthcare provision between groups from different socioeconomic status. Consideration of a cause of death as amenable was based on the existence of effective interventions to prevent it. The results of this study show that amenable mortality makes a significant contribution to general mortality (around 10%, higher among men), having decreased over time in the three cities studied for men and women. Results are consistent, since in the three cities studied the risks of death are higher in areas of greater deprivation, and these excesses have not modified over time.

The percentage of amenable deaths with regard to general mortality has been decreasing over the three study periods, for men and women, in the three cities studied. The decrease observed for amenable mortality over time agrees with the results obtained in other studies suggesting that improvements in health services have contributed significantly to the improvement of the population’s health in recent years [11,18,19,23,26,28,42]. The decreasing trend in amenable mortality found in this study is similar to that found for preventable mortality in Spain in other studies [43,44].

The socioeconomic inequalities found are along the same lines as those observed in other studies. For example, Tobias and Yeh found that amenable causes make an important contribution to the mortality differentials between socio-economic groups in New Zealand, with data from 1981–84 and 2001–04 [8]. Furthermore, Nagy, et al., examining data from 2004 to 2008, found a relationship between the risks of death due to amenable causes and deprivation in geographical areas of Hungary [22]. Schoenbaum, et al. provided evidence of excessive amenable mortality in areas with higher populations of poor and black people in the US in 2005 [21]. In the city of New York, Althoff, et al. found that disparities in the mortality due to several amenable causes of poor and wealthy have not narrowed between 1989–91 and 1999–2001 [45]. Using data from 14 European countries (including Spain for the city of Barcelona and the regions of Madrid and the Basque Country), Plug, et al. observed the presence of inequalities in amenable mortality regarding level of education [44].

In relation to the evolution of socioeconomic inequalities over time, this study does not find evidence of modification between the periods studied with regard to inequalities between socioeconomic status levels in any of the three cities, since the effects of interaction between SES and the study period were not significant, possibly indicating that inequalities have remained constant in both periods. This result coincides with the findings of some studies but counters the results of others. Korda, et al., looking at small areas of Australia, observed that amenable mortality decreased significantly but more so among the highest levels of SES between 1986 and 2002 [18]. James, et al. observed a reduction in socioeconomic differences in amenable mortality in Canada between 1971 and 1996, maintaining significant inequalities [26]. In Finland, Lumme, et al. observed, using data from 1992 to 2008, that in spite of the decrease in amenable mortality rates, socioeconomic differences have increased, coinciding with the findings of McCallum, et al. for that same country [23,30]. In Canada, James, et al. found a significant decrease in socioeconomic inequalities in amenable mortality in urban areas, between 1971 and 1996, although this was not the same for preventable mortality owing to public health policies [19].

The findings of this study can be compared with those obtained by the authors in a previous study conducted on the same cities, with the same socioeconomic indicators, census tracts, and a similar methodology to estimate socioeconomic inequalities in preventable avoidable mortality with data from 1996 to 2003 [34]. In that research, higher risks of death were found owing to causes of preventable mortality in the most underprivileged areas, but they were greater than those obtained here for amenable mortality among men (relative risks above 2 in the three cities), and lower among women (only the city of Valencia presented a value significantly higher than 1). However, the results obtained from this study point to excesses in the risk of death in areas with a lower socioeconomic status that are similar among men and women, and in all three cities, suggesting that the effect of socioeconomic inequalities on amenable mortality is more homogenous than on preventable mortality. The interaction detected between the effect of SES and the effect of age among women in the city of Valencia nuances the interpretation of excess mortality among more under privileged SES levels, establishing a significant excess risk only among older women (>50 years of age).

In relation to the classification by SES used in this work, it is worth mentioning that the relative risks estimated between the most and least privileged categories represent the relative risk between the worst and best-off population group in all the indicators used. Hence, the interpretation of these relative risks differs from that obtained using other classifications based on percentiles or on the continuous value of a compound socioeconomic indicator based on the originals, and indicates the level of extreme inequalities, between categories that can be identified as being of maximum and minimum deprivation. This classification identifies areas of maximum deprivation, susceptible to greater vigilance and attention.

The most common cause of amenable mortality in this study for men was ischaemic heart disease, although only 50% of deaths were included, which was also the third most frequent cause of death for women. In a recent study conducted in small areas of nine major cities in Spain, a decrease in mortality owing to this cause was found over time in small areas of Spanish cities between 1996 and 2007, coinciding with the decrease in amenable mortality found here [46]. The socioeconomic inequalities found in this study for this cause were greater among women than among men, which cannot be firmly asserted for amenable mortality studied as a whole in this article since, although the relative risks of death estimated between levels of SES are slightly higher among women, they do not present significant differences with men, which suggests the socioeconomic inequalities in amenable mortality are similar in the two sexes.

Amenable mortality has been used for decades to assess health care delivery. Recent studies have confirmed that such indicator is useful to establish the impact of health care innovation on population’s health. However, they suggest that amenable mortality should be interpreted with caution when being used as an indicator of health care performance, especially when comparing different countries, for this indicator may reflect the influence of health care and other factors [36,47,48]. Our study confirms, on the one hand, the idea that amenable mortality is related not only to health care delivery but also to other factors. Socioeconomic inequalities found show not only an unequal health care delivery but also many other unequal socioeconomic factors that influence mortality. On the other hand, this study has been performed in urban areas, where the overall population has health insurance and medical coverage. Furthermore, the three cities have a common health care policy framework, and hence, the innovations and improvements were introduced at the same time for the whole population. These conditions suggest that health care delivery may have an independent effect on amenable mortality, which may not confound the effect of other factors that belong to socioeconomic status because of the conditions described above (overall medical coverage and health insurance, urban areas, similar policy framework…), which make it difficult to think about an association between socioeconomic factors and health care delivery. These conditions are different to the ones in the study of Shoenbaum et al., carried out on United States population with different health insurance and medical coverage. This study described the association between amenable mortality and indicators related to both health care and socioeconomic factors, and concluded that it is important to adjust the effect of one on the other [21].

The causes of inequality observed between socioeconomic status levels can be complex.

On the one hand, the differences found might pertain to different access to and quality of healthcare according to SES, as suggested in various studies, so that higher income population segments are more likely to see a specialist [49,50]. However, other studies have not found any links between inequalities in the use of healthcare and amenable mortality [44]. On the other, the prevalence, incidence, and natural course of some diseases might have an effect on amenable mortality and differ between socioeconomic groups, since the exposure to its risk factors might also differ. Survival following treatment might also be affected by different resources according to socioeconomic status (social support, resources at home, medical insurance, etc.). These possible differences have not been taken into account in this study. Regardless of accessibility and the quality of services, people’s socioeconomic status will be related with their level of culture, and can influence aspects such as self-care, and access to information about the health-disease process, factors that influence the way in which healthcare services are accessed and used. Hence, for many amenable causes, there is access to healthcare services but they are not used in the early stages of the diseases; people reach the healthcare system when their condition has worsened, and there might be a direct influence between the patient’s trajectory of self-care and mortality.

This study was conducted during a period in which the Spanish Healthcare System offers universal and free access to all citizens, differentiating it from other countries where this is not the case. This starting point is favourable to reducing mortality and to achieve equity but does not guarantee it, as shown by the results of this and other studies [37,51,52].

This study presents certain limitations. Firstly, it is an ecological study, with all the limitations this kind of study brings. Thus, it does not allow the proof of a causal association. The association found between SES and mortality using CTs may not be applicable at an individual level (i.e. ecological fallacy). With reference to the causes analysed, the list used could have differed. The choice of these causes responded to a criterion of comparability with many other studies. The consistency of the results obtained indicates that they would not have been substantially different from those obtained using a different list with minor variations. Hence, the inclusion of 50% of deaths due to ischaemic heart disease was revised, recalculating the relative risks between levels of SES with 100% of deaths, but no substantial variations were found in the estimations. Another limitation could pertain to the use of different classifications of mortality over the period of study, which were felt to be minimal, given the experience of the encoding team from the Death Register Office, which was the same throughout the period of study. The classification of census tracts by SES was carried out using accumulated data from the 2001 census and was the same for the entire period of study. The year 2001 is right in the middle of the study period and it was thought that there would not be substantial variations over the course of this period. Another aspect to consider is that avoidable mortality only takes account of deaths and no other health results, offering an incomplete vision of the benefits of healthcare. Furthermore, as mentioned previously, the causes of socioeconomic differences in amenable mortality can come from a complex model in which differences in the prevalence or incidence of diseases are involved, along with differences in pre and post treatment resources, and even biases in access to and quality of healthcare.

Inequalities in health, and specifically the differences reflected in amenable mortality, which emerge between the least and the more privileged groups in the population, is undoubtedly one of the greatest challenges faced by healthcare policies and modern healthcare systems. The differences in avoidable mortality, preventable or amenable, are usually the reflection of an unequal system, and their analysis is essential in order to understand in detail the weaknesses of the system, and to take effective actions.

The period studied in this article concludes in 2007, coinciding with the start of the current global economic crisis that is having such a major effect on Spain. As such, this work could provide a point of reference for future studies that assess the evolution of inequalities in subsequent periods.

## Conclusions

This study consistently highlights that, although amenable mortality decreases over time, its socioeconomic inequalities are maintained in the three cities. Areas have been identified that show excesses in amenable mortality, potentially attributable to deficiencies in the healthcare system, and associated with low socioeconomic level. These are the higher risk areas where action must be taken to reduce inequalities in health detected. It is necessary, therefore, to study in depth the causes of socioeconomic inequalities in amenable mortality.

## Abbreviations

CV: Comunitat Valenciana; ICD-9: International classification of diseases, 9th version; ICD-10: International classification of diseases, 10th version; CT: Census tract; SES: Socioeconomic status; SES1: Most privileged socioeconomic status; SES2: Intermediate socioeconomic status; SES1: Least privileged socioeconomic status; P1: Time period 1996–1999; P2: Time period 2000–2003; P3: Time period 2004–2007; RR: Relative risk; IHD: Ischaemic heart disease.

## Competing interests

The authors declare that they have no competing interests.

## Authors’ contributions

The study was conceived and designed collectively by all the authors. AN and JAQ coordinated and conducted the analysis and wrote the first version of the manuscript. All the authors contributed equally to the analysis of the data, interpretation, and discussion of results. All the authors have read and approved the final version.

## Pre-publication history

The pre-publication history for this paper can be accessed here:

http://www.biomedcentral.com/1471-2458/14/299/prepub
